# Health Tract No. 342

**Published:** 1868-10

**Authors:** 


HEALTH TRACT NO. 342.

### LUNACY.

How we shrink with horror that we ourselves or children should die in a mad house! yet it is a preventable event, in our own power, in nine cases out of ten. From a number of reports of different asylums, some general deductions of great practical importance may be safely and logically drawn.

1st. Nearly one half of all who are treated by the most approved methods of the present time, to wit, kindness, unrestraint and agreeable occupation, are permantly cured.

2d. Every day that passes after persons have been observed to be insane, without sending them to a competent asylum, increases the chances of incurability; hence it is a mischievous delicacy and false pride, which delays their being sent, in the indefinite hope that they may get well without it, as very few ever recover who are detained at home over a year after the first symptoms have been noted; nine-tenths of the recoveries being of those who were sent within the first year.

3d. More women are sent than men.

4th. More single persons are sent than married.

5th. A greater number of women in proportion to those sent, get well than men; first because they have rest; second, indoor life is not so wearing upon them as men, being more accustomed to it.

6th. More than one fourth of all the inmates are from the families of farmers and merchants, not wholly because they constitute the largest classes of the community; so many of the former, not because the occupation itself is not healthful, but for want of mental culture and their ignorance and inattention to the laws of their being; of the latter, because they grow up as the "elite" of the communities in which they live, but later on become so often bankrupt, the mind fails in the attempt to grapple with the difficulties of their changed condition.

7th. Ill health is the most prominent cause of insanity, induced by insufficent exercise, intemperance, over-eating and yielding to trouble, care and mental anxiety; the always certain remedy against these being a more general cultivation of out-door activities, a greater attention to stirring business, giving preference to those occupations which are congenial, absorbing and encouragingly remunerative.

